# Legumain is a paracrine regulator of osteoblast differentiation and mediates the inhibitory effect of TGF-β1 on osteoblast maturation

**DOI:** 10.3389/fendo.2024.1445049

**Published:** 2024-09-19

**Authors:** Karl Martin Forbord, Ngoc Nguyen Lunde, Tatjana Bosnjak-Olsen, Harald Thidemann Johansen, Rigmor Solberg, Abbas Jafari

**Affiliations:** ^1^ Section for Pharmacology and Pharmaceutical Biosciences, Department of Pharmacy, University of Oslo, Oslo, Norway; ^2^ Department of Cellular and Molecular Medicine, University of Copenhagen, Copenhagen, Denmark

**Keywords:** legumain, asparaginyl endopeptidase, transforming growth factor beta 1, matrix mineralization, RR-11a analog

## Abstract

Transforming growth factor-beta 1 (TGF-β1) is a critical regulator of skeletal homeostasis and has diverse effects on osteoblastogenesis. To date, the mechanisms behind the intriguing inhibitory effect of TGF-β1 on osteoblast maturation are not fully understood. Here, we demonstrate a novel mechanism by which TGF-β1 modulates osteoblast maturation through the lysosomal protease legumain. We observed that addition of TGF-β1 to osteogenic cultures of human bone marrow derived mesenchymal stromal (stem) cells enhanced legumain activity and secretion, in-spite of decreased legumain mRNA expression, suggesting post-transcriptional regulation. We further showed that osteogenic cells internalize and activate prolegumain, associated with inhibited osteoblast maturation, revealing legumain as a paracrine regulator of osteoblast maturation. Interestingly, TGF-β1 treatment exacerbated legumain internalization and activity, and showed an additive effect on legumain-induced inhibition of osteoblast maturation. Importantly, pharmacological inhibition of legumain abolished the inhibitory effect of TGF-β1 on osteoblast maturation. Our findings reveal that TGF-β1 inhibits osteoblast maturation by stimulating secretion and activity of endogenous legumain, as well as enhancing internalization and activation of extracellular prolegumain. Therefore, our study provides a deeper understanding of the complex regulation of osteoblastogenesis and unveils a novel TGF-β1-legumain axis in regulation of osteoblast maturation, offering novel insights for possible therapeutic interventions related to bone diseases associated with aberrant TGF-β1 signaling.

## 1 Introduction

Transforming growth factor-beta 1 (TGF-β1) is one of the most abundant cytokines in the bone matrix (1, 2). This multifunctional cytokine plays a key role in regulation of skeletal homeostasis by orchestrating various cellular processes involved in bone development, remodeling, and repair, which are elucidated by compelling evidence from human and mouse studies (3). Domain-specific heterozygous mutations in human TGF-β1 are linked to Camurati-Engelmann disease; an autosomal dominant disease associated with increased bone remodeling and osteosclerotic lesions in the long bones and skull (4). TGF-β1 deficiency in mice leads to early death due to organ failure associated with autoimmune disease (5). Analysis of the tibiae from TGF-β1 deficient mice exhibited decreased longitudinal growth and bone mineral content (6). In addition, histological analysis of bones from TGF-β1 deficient mice indicated depletion of pre-osteoblasts at the trabecular bone surfaces and the presence of osteoprogenitors in the middle of the bone marrow (7). At the cellular level, TGF-β1 has been shown to enhance osteoblast proliferation (8) and migration of osteogenic cells to the bone forming surfaces (9), while exerting an inhibitory effect on osteoblast apoptosis (10). Furthermore, TGF-β1 has also been shown to enhance commitment of bone marrow stromal cells to the osteogenic lineage (i.e. early-stage osteoblast differentiation) (3). In contrast, TGF-β1 also exerts an inhibitory effect on osteoblast maturation (i.e. late-stage osteoblast differentiation) (11). Although the mechanisms behind pro-osteogenic actions of TGF-β1 are well-established and have been shown to be mediated through e.g. stimulating the expression of master regulators of osteogenesis, such as Runx2 and osterix/Sp7 (13), the mechanism responsible for the intriguing inhibitory effect of TGF-β1 on osteoblast maturation are not well understood.

Legumain (also known as asparaginyl endopeptidase) is a lysosomal cysteine protease involved in protein activation, processing, and degradation, thereby playing roles in diverse biological processes [reviewed in (14)]. Legumain is also secreted and is measurable in body fluids such as serum/plasma and is thus postulated to have autocrine/paracrine/endocrine functions [reviewed in (15)]. In addition, we have previously shown that high levels of legumain is present in the bone microenvironment (16). Furthermore, we showed that legumain inhibits osteoblast maturation of bone marrow mesenchymal stromal (stem) cells (BMSC) and thus bone formation through degradation of the extracellular matrix protein fibronectin (16).

Herein, we provide evidence for a novel cross-regulatory function of TGF-β1 and legumain, in which TGF-β1 enhances legumain activity, secretion, and activation of internalized extracellular prolegumain, and in return, legumain mediates the inhibitory effect of TGF-β1 on osteoblast maturation. Our findings not only advance our understanding of the complex regulatory networks governing osteoblast function but also provide novel insight into the mode of action of TGF-β1 in regulation of skeletal homeostasis. Thus, our studies may unveil potential therapeutic avenues for bone-related disorders mediated by aberrant TGF-β1 signaling.

## 2 Materials and methods

### 2.1 Cell culturing and osteogenic differentiation

Human bone marrow derived mesenchymal stromal (stem) cells (hBMSC) overexpressing the human telomerase reverse transcriptase (hBMSC-TERT, RRID: CVCL_Z017; (17); hereby referred to as BMSC) were used and cultured in basal medium containing Minimal Essential Media (MEM) with L-glutamine, 10% (v/v) fetal bovine serum (FBS), 1% penicillin (100 U/mL) and streptomycin (100 µg/mL). Human embryonic kidney 293 (HEK293; American Type Culture Collection (ATCC), Rockville, USA, RRID: CVCL_0045) and monoclonal legumain over‐expressing HEK293 (M38L) cells were cultured in Dulbecco’s Modified Eagle Medium (DMEM) with 1 mM sodium pyruvate, 10% FBS, 100 U/ml penicillin 100 μg/ml streptomycin with (M38L) or without (HEK293) 800 μg/ml G418 (Sigma-Aldrich) as previously described (18). For production of conditioned media (CM), HEK293 and M38L cells were seeded at a density of 5*10^4^ cells/cm^2^ in serum-free media, maintained at 37°C and 5% CO_2_ in a humidified atmosphere before CM were collected after 4 days and used as reagents for treatment of BMSC.

For osteogenic differentiation, BMSC cells were seeded at a density of 2*10^4^ cells/cm^2^ and at 80% confluence, the cells were incubated for 14 days in osteogenic induction medium (OBIM) (16), consisting of basal medium supplemented with 50 µg/mL L-ascorbic acid, 10 mM β-glycerophosphate, 10 nM 1,25-dihydroxyvitamin D_3_, 10 nM dexamethasone and 10% (v/v) FBS. For osteogenic differentiation in prolegumain rich CM, BMSC were incubated for 3, 7 or 14 days in OBIM with double the concentrations of the abovementioned ingredients (2xOBIM; including FBS to compensate for the serum-free CM), diluted 1:1 with CM from M38L (prolegumain concentration 165 ng/mL; measured by ELISA) or HEK293 (control, prolegumain concentration 1.1 ng/mL; ELISA) cells. In addition, the BMSC cells were incubated with or without TGF-β1 (25 ng/mL) (R&D systems, MN, USA) and/or 50 µM of the irreversible legumain inhibitor RR-11a analog (referred as RR-11a; MedChemExpress, NJ, USA). An equal volume of solvent (4 nM HCl and 3% bovine serum albumin in distilled H_2_O (dH_2_O) for TGF- β1 and DMSO for RR-11a) was used as control. The medium was changed every 3-4 days.

### 2.2 Harvesting of conditioned media and cell lysates

Cell conditioned media were collected, centrifuged at 800 rpm for 10 minutes at 4°C and frozen at -20°C. Adherent cells were washed with PBS before adding legumain lysis buffer (100 mM sodium citrate, 1 mM disodium-EDTA, 1% n-octyl-β-D-glucopyranoside, pH 5.8) or Buffer RLT Pluss buffer (QIAGEN, Hilden, Germany). Cell lysates were sonicated for 20 seconds and centrifuged at 10 000 g for 5 minutes before the supernatants were frozen at -20°C (in legumain lysis buffer), -70°C (in Buffer RLT) or directly analyzed. Total protein concentration in cell lysates was measured at 595 nm according to (19) and the manufacturer (Bio-Rad Laboratories, Hercules, CA, USA) in a microplate reader (Wallac Victor^®^ Nivo™, Perkin Elmer, Boston, MA, USA). Bovine serum albumin (0-400 µg/mL) was used as a standard for calculation of total protein concentrations. All measurements were performed in triplicates.

### 2.3 Immunoblotting and enzyme-linked immunosorbent assay

Gel electrophoresis and immunoblotting were performed by applying 15 µg total protein using NuPAGE 4-12% gels (Life Technologies) and the supplied NuPAGE MOPS SDS running buffer, prior to transfer to a nitrocellulose membrane (Trans-Blot^®^ Turbo™ Mini-size nitrocellulose) in the Trans-Blot^®^ Turbo™ Transfer System for 30 minutes. The membranes were blocked for 1 hour at room temperature with Odyssey^®^ Blocking Buffer and probed with goat polyclonal anti-legumain (1:200, AF2199, R&D systems, RRID: AB_416565) or mouse monoclonal anti-GAPDH (1:10 000, MAB5718, R&D systems, RRID: AB_10892505) antibody in blocking buffer/0.2% T-TBS (Tween 20 in TBS; 1:1) overnight at 4°C. Membranes were subsequently washed 3-4 times in T-TBS buffer and incubated with donkey anti-goat IR Dye 680LT (1:10 000, LI-COR, Cambridge, UK) or donkey anti-mouse 800CW (1:10 000, LI-COR) for 1 hour at room temperature. After another washing procedure, membranes were briefly dried and analyzed using Odyssey-CLx Imaging System (LI-COR). Immunoband intensities were quantified using the Image Studio Lite 5.2 software (LI-COR). Legumain concentration in conditioned media was measured using a total legumain ELISA kit (DY4769; R&D Systems, RRID: AB_294369) according to the manufacturer’s protocol.

### 2.4 Legumain activity assay

Cleavage of the peptide substrate Z-Ala-Ala-Asn-AMC (Bachem, Bubendorf, Switzerland) was used to measure the proteolytic activity of legumain in cell lysates as previously described (20). In brief, 20 µL sample, 100 µL assay buffer (39.5 mM citric acid, 121 mM Na_2_HPO_4_, 1 mM Na_2_EDTA, 0.1% CHAPS, pH 5.8 and 1 mM DTT), 5 µL of the pan-cathepsin protease inhibitor E64 (to abolish unknown asparagine endopeptidase activity, 1 µM final concentration, Sigma Aldrich, MA, USA) and 50 µL peptide substrate solution (final concentration 10 µM) were added in black 96-well microtiter plates (Corning Life Science, MA, USA). Kinetic measurements based on increase in fluorescence (360EX/460EM) over 10 or 60 minutes were performed at 30°C in Victor^®^ Nivo™ microplate reader. Kinetics were calculated as peak increase in fluorescence per second (dF/sec) and corrected for total protein concentration (µg/mL) in the sample. Negative activity values were shown as zero activity in the respective figures, although statistics was calculated against the actual values.

### 2.5 Quantitative PCR

Total RNA was extracted and purified from cell lysates harvested in Buffer RLT Pluss using a RNeasy^®^ Plus Kit (QIAGEN) according to the manufacturers protocol. Complement DNA (cDNA) was synthesized using High-Capacity cDNA Reverse Transcription Kit (Applied Biosystems, Warrington, UK) and TaqMan Reverse Transcription Reagents (Applied Biosystems) in a PerkinElmer 2720 Thermal Cycler (PerkinElmer, Shelton, CT, USA) (25°C for 10 min, 37°C for 90 min, 85°C for 5 min). RT-qPCR was performed using PowerTrack SYBR Green Master Mix (Applied Biosystems) and the Applied Biosystems StepOnePlus™ Instrument with the accompanying software StepOne™. β-actin forward (5’-3’: ACC GAG CGC GGC TAC A) and reverse primer sequence (5’-3’: TCC TTA ATG TCA CGC ACG ATT T). GAPDH forward (5’-3’: GTC TCC TCT GAC TTC AAC AGC G) and reverse primer sequence (5’-3’: ACC ACC CTG TTG CTG TAG CCA A). Legumain forward (5’-3’: GCA GGT TCA AAT GGC TGG TAT) and reverse primer sequence (5’-3’: GGA GTG GGA TTG TCT TCA GAG T). ΔCT was calculated as the CT value of the gene of interest, minus the mean of CT values of housekeeping genes (β-actin and GAPDH). ΔΔCT was calculated as ΔCT, minus the average ΔCT of control samples.

### 2.6 Quantification of matrix mineralization

Matrix mineralization was quantified using alizarin red staining as previously described (21). In brief, the conditioned medium was removed before the cells were washed briefly in PBS and fixed in 70% ice-cold ethanol at -20°C for one hour. The fixed cells were rinsed in dH2O and stained for 10 minutes with tilting in 40 mM aqueous alizarin red solution (pH 4.2) at room temperature. Excess dye was removed with dH2O, followed by three washes in PBS to reduce nonspecific staining. The stained cells were scanned using a photo scanner (Epson Perfection V600, Epson, Suwa, Japan) before alizarin red was eluted in an aqueous 20% methanol and 10% acetic acid solution. Aliquotes (100 µL) of the eluate was added in triplicates to a 96-well plate and absorbance was measured at 570 nm in a microplate reader (Wallac Victor^®^ Nivo™, Perkin Elmer).

Matrix mineralization was also quantified using IRDye^®^ 800CW BoneTag™ (LI-COR, Cambridge, UK) as previously described (22). In brief, BoneTag™ (final concentration 2 pmol/mL) was added to the cell culture medium the day before analysis. After 24 hours, the conditioned medium was removed, and the cells were washed with PBS. Fresh PBS was added, and fluorescence was measured at 800 nm using the Odyssey-CLx Imaging System (LI-COR) and quantified using the Image Studio Lite 5.2 software.

### 2.7 Statistics

Data is presented as mean ± standard error of mean (SEM). Two-tailed Students t-test, two-way, or three-way ANOVA was performed when appropriate. Statistical significance was considered at p<0.05. Calculations were performed using GraphPad Prism (V9.0; GraphPad Software, Inc., San Diego, CA, USA) or R Statistical Software [V4.2.2; (23)].

## 3 Results

### 3.1 TGF-β1 inhibits formation of mineralized matrix by osteogenic BMSC cultures

**Figure 1:**
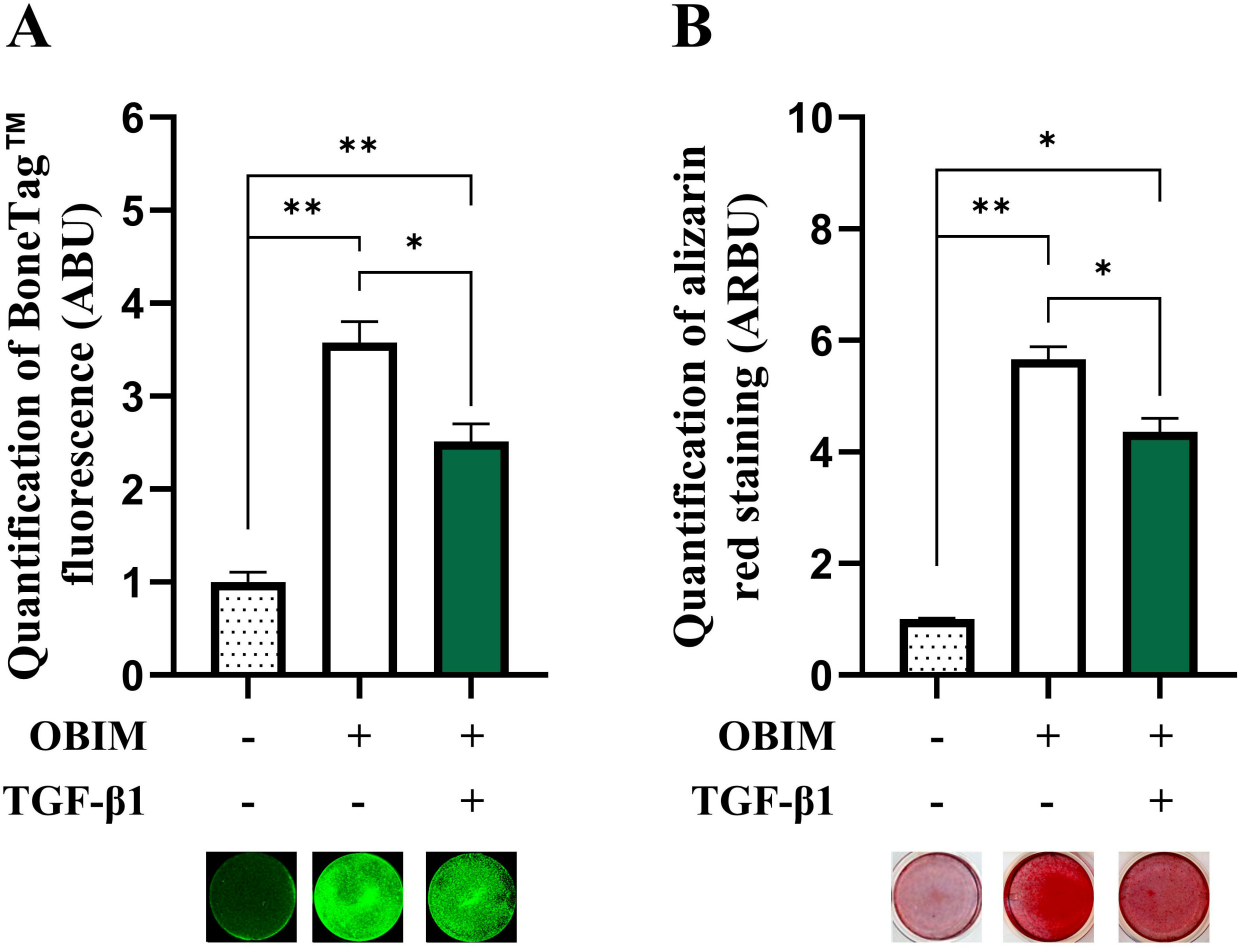
TGF-β1 inhibits mineralized matrix formation in osteogenic BMSC cultures. BMSC were cultured in basal, osteogenic induction medium (OBIM) or OBIM with TGF-β1 (25 ng/mL) for 14 days. Matrix mineralization was measured by BoneTag™ fluorescence [**(A)** n=3-4] and alizarin red staining [**(B)** n=3]. Normalized data represent mean ± SEM. Two-way ANOVA (Šidák correction). *p<0.05, **p<0.01. Numbers (n) represent individual biological replicates.

To investigate the mechanism(s) involved in TGF-β1-induced inhibition of osteoblast maturation, we employed osteogenic cultures derived BMSCs. To validate the suitability of this cell model for our studies, we first determined the effect of the osteogenic induction medium (OBIM) on osteoblast maturation and formation of mineralized matrix by cultivating BMSCs in basal medium or OBIM for 14 days. In addition, to determine the effect of TGF-β1, cells cultured in OBIM were also treated with or without TGF-β1 (25 ng/mL). The formation of mineralized matrix was quantified using BoneTag™ (**Figure 1A**) and alizarin red staining (**Figure 1B**). Both methods indicated a clear increase in matrix mineralization in cells cultured in OBIM compared to basal medium. In addition, TGF-β1 had a significant inhibitory effect on osteoblast maturation in cells cultured in OBIM.

### 3.2 TGF-β1 enhances legumain activity and secretion, but not mRNA expression in osteogenic BMSC cultures

**Figure 2:**
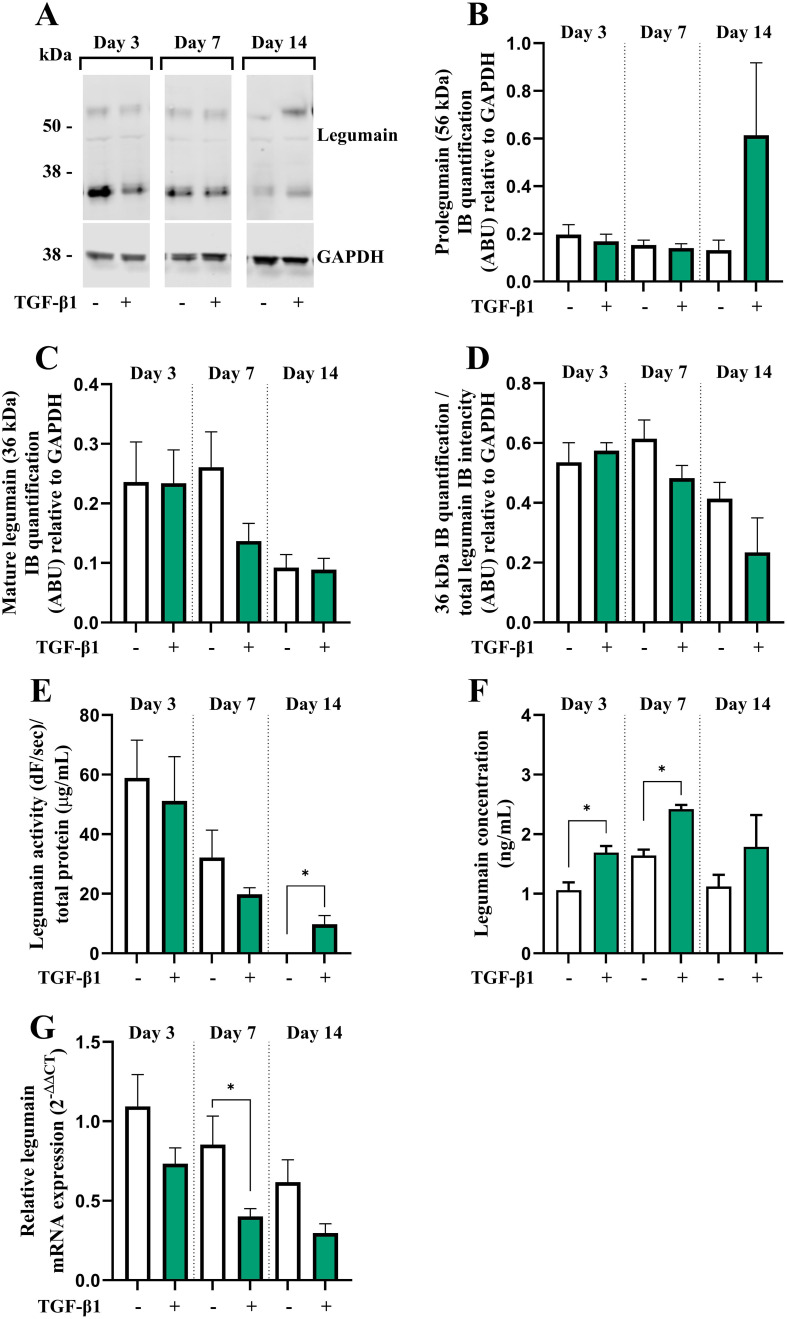
TGF-β1 enhances legumain activity and secretion, but not mRNA expression in osteogenic BMSC cultures. BMSC were cultured in 2x osteogenic induction medium diluted 1:1 with conditioned control medium (HEK293-CM) with or without TGF-β1 (25 ng/mL) for 3, 7 or 14 days. **(A)** One representative immunoblot of legumain and GAPDH (housekeeping) in cell lysates (n=4). **(B, C)** Quantification of the 56 kDa [**(B)** prolegumain] and 36 kDa [**(C)** mature legumain] immunoband (IB) intensities as arbitrary units (ARBU) relative to GAPDH on immunoblots presented in **(A)** (n=4). **(D)** Mature legumain fraction, calculated as the 36 kDa IB intensities normalized to the total legumain band intensities (36 kDa + 56 kDa) as ARBU relative to GAPDH on immunoblots presented in **(A)** (n=4). **(E)** Legumain activity (dF/sec) in cell lysates adjusted for total protein concentration (µg/mL) (n=3). **(F)** Legumain concentration (ng/mL) in conditioned media measured by ELISA (n=6). **(G)** Legumain mRNA expression relative to the mean CT values of two housekeeping controls (GAPDH and β-actin) (2^-ΔΔCT^; n=4-6). **(B–F)** Data represent mean ± SEM. **(B–F)** Two-tailed students t-test. **(G)** Two-tailed students t-test on ΔCT values. *p<0.05. Numbers (n) represent individual biological replicates.

We have reported legumain as a novel inhibitor of osteoblast maturation (16). To assess whether the inhibitory effect of TGF-β1 could be mediated through altered legumain expression and function in osteogenic cultures, legumain levels were determined in BMSC cultures in the presence or absence of TGF-β1 (25 ng/mL) during osteogenic differentiation for up to 14 days. Immunoblot analysis of cell lysates from osteogenic cultures on day 3, 7, and 14 of differentiation showed that TGF-β1 did not significantly affect protein expression of either pro- or mature legumain at any time point, although a tendency towards increased levels of prolegumain was observed on day 14 of differentiation (**Figures 2A–C**). The mature legumain fraction was also unaffected (**Figure 2D**). We then determined the effect of TGF-β1 on the activity of endogenous legumain in osteogenic cultures and observed increased legumain activity in cells cultured with TGF-β1 for 14 days (**Figure 2E**). In addition, treatment with TGF-β1 increased the concentration of legumain in the conditioned media at day 3 and 7, indicating increased legumain secretion (**Figure 2F**). In contrast, legumain mRNA expression was decreased by TGF-β1, reaching significance at day 7 of osteogenic differentiation (**Figure 2G**).

### 3.3 TGF-β1 enhances activation of internalized prolegumain during osteogenic differentiation of BMSC

**Figure 3:**
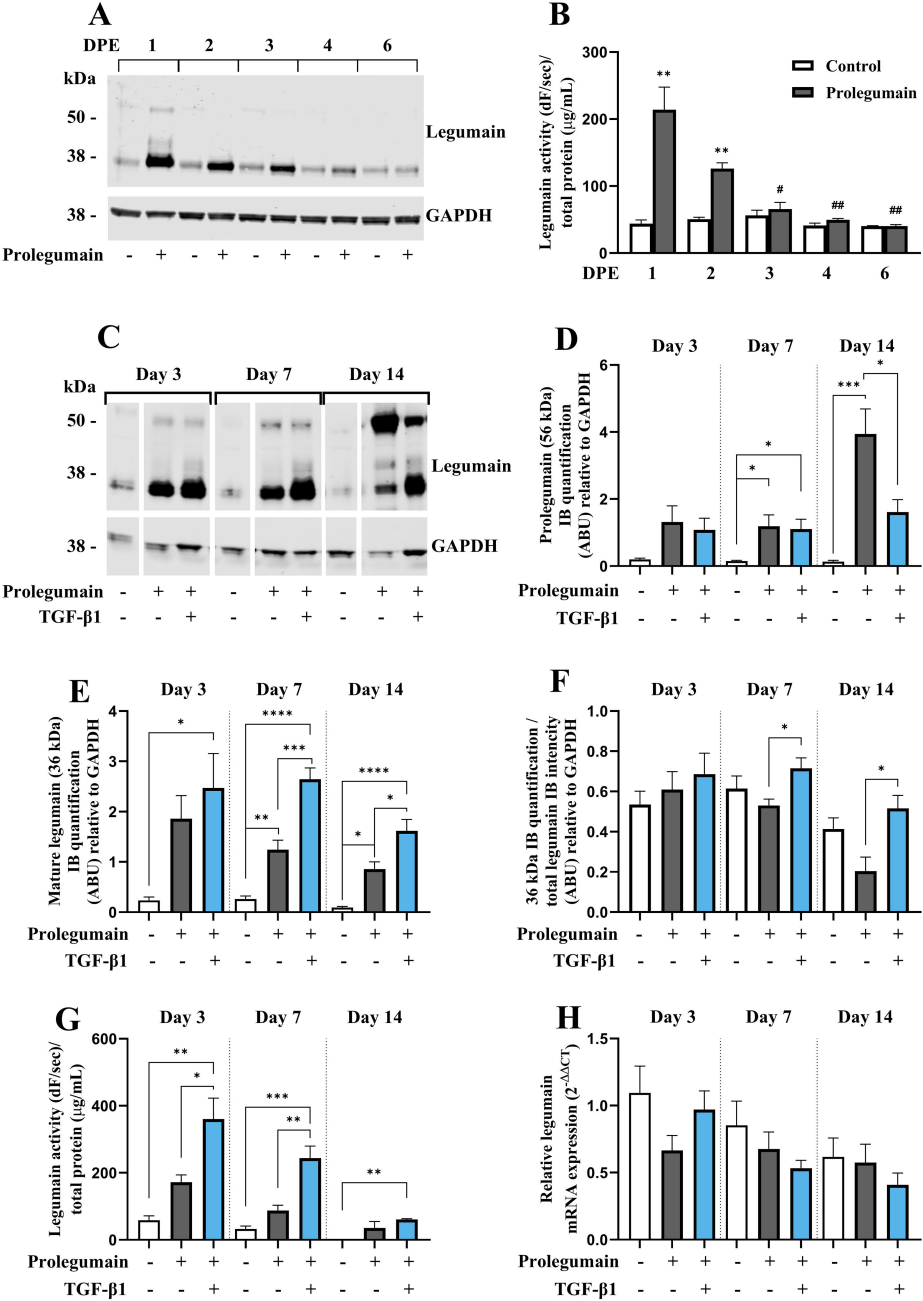
TGF-β1 enhances activation of internalized prolegumain during osteogenic differentiation. **(A, B)** BMSC were cultured in basal medium with 20% FBS with or without treatment with 50% prolegumain-rich conditioned medium (165 ng/ml, control: 1.1 ng/mL)for 24 hours before the medium was changed to basal medium. Lysates were harvested 1, 2, 3, 4 and 6 days post exposure (DPE). **(C–G)** BMSC were differentiated to osteogenic cells for 14 days with or without treatment with 50% prolegumain-rich conditioned medium (165 ng/mL, control: 1.1 ng/mL) and with or without TGF-β1 (25 ng/mL) for 3, 7 or 14 days. **(A, C)** One representative immunoblot of legumain and GAPDH (housekeeping) in cell lysates (n=3-4). **(D, E)** Quantification of the 56 kDa [**(D)**; prolegumain] and 36 kDa [**(E)**; mature legumain] immunoband (IB) intensities as arbitrary units (ARBU) relative to GAPDH on immunoblots presented in **(C)** (n=4). **(F)** Mature legumain fraction, calculated as the 36 kDa IB intensities normalized to the total legumain band intensities (36 kDa + 56 kDa) as ARBU relative to GAPDH on immunoblots presented in **(C)** (n=4). **(B, G)** Legumain activity (dF/sec) in cell lysates adjusted for total protein concentration (µg/mL) (n=3). **(H)** Legumain mRNA expression relative to the mean of CT values of two housekeeping controls (GAPDH and β-actin) (2^-ΔΔCT^; n=4-6). **(B, D–H)** Data represent mean ± SEM. **(B)** Two-tailed students t-test. **(D–G)** Two-way ANOVA (Šidák correction). **(H)** Two-way ANOVA (Šidák correction) on ΔCT values. *p<0.05, **p<0.01, ***p<0.001, ****p<0.0001 [**(B)** compared to control on same DPE]. #p<0.05, ##p<0.01 compared to same treatment on 1 DPE. Numbers (n) represent individual biological replicates.

Prolegumain has previously been shown to be secreted by various cell types, to be present in extracellular environments (e.g. plasma) and to be internalized and activated by recipient cells (15, 18). Since we demonstrated that legumain was secreted during osteogenic differentiation and that the secretion was enhanced by TGF-β1, we aimed to investigate whether prolegumain could be internalized and activated by BMSC. The cells were incubated in a 1:1 mixture of basal medium and conditioned media from HEK293 cells overexpressing and secreting legumain [prolegumain concentration 165 ng/mL; (18)] or HEK293 control cells (control; prolegumain concentration 1.1 ng/mL) for 24 hours before the media was switched to fresh basal medium. Cell lysates were harvested daily for 6 days following the single, 24 hours prolegumain exposure. Immunoblot analysis of cell lysates showed that prolegumain (56 kDa) was internalized and almost completely processed to mature legumain (36 kDa) 24 hours after exposure to extracellular prolegumain, whereas the legumain level returned to baseline after 6 days (**Figure 3A**). We also found significantly increased legumain activity in BMSC lysates 1 and 2 days post exposure (DPE) to prolegumain, whereas the initially increased legumain activity returned to baseline after 3 days (**Figure 3B**).

Next, we investigated whether prolegumain could be internalized by osteogenic cells at different stages of differentiation, and if TGF-β1 affected legumain internalization and activation. BMSC were cultured in osteogenic induction medium with or without prolegumain-rich medium and with or without TGF-β1 (25 ng/mL) for 3, 7 or 14 days. Immunoblot analysis of cell lysates revealed significantly increased level of prolegumain (56 kDa) at day 7 and 14, whereas mature legumain (36 kDa) was increased at all time points upon exposure to prolegumain, supporting internalization and processing of prolegumain in osteogenic cultures (**Figures 3C–E**). Interestingly, concomitant treatment with prolegumain and TGF-β1 significantly reduced the intracellular level of prolegumain at day 14. In contrast, increased level of mature legumain and mature legumain fraction (**Figure 3F**) was observed at day 7 and 14 by the combined treatment with TGF-β1 and prolegumain, compared to prolegumain treatment alone. In addition, we observed a tendency towards increased legumain activity in osteogenic cultures exposed to extracellular prolegumain, whereas combined treatment with TGF-β1 and prolegumain significantly increased legumain activity on day 3 and 7 of osteoblast differentiation, compared to prolegumain treatment alone (**Figure 3G**). We did not observe any changes in legumain mRNA expression upon exposure of osteogenic cultures to extracellular legumain and/or TGF-β1 (**Figure 3H**). In summary, these results indicated that TGF-β1 promotes processing and activation of internalized legumain.

### 3.4 Legumain inhibits osteoblast maturation through a paracrine mechanism exacerbated by TGF-β1

**Figure 4:**
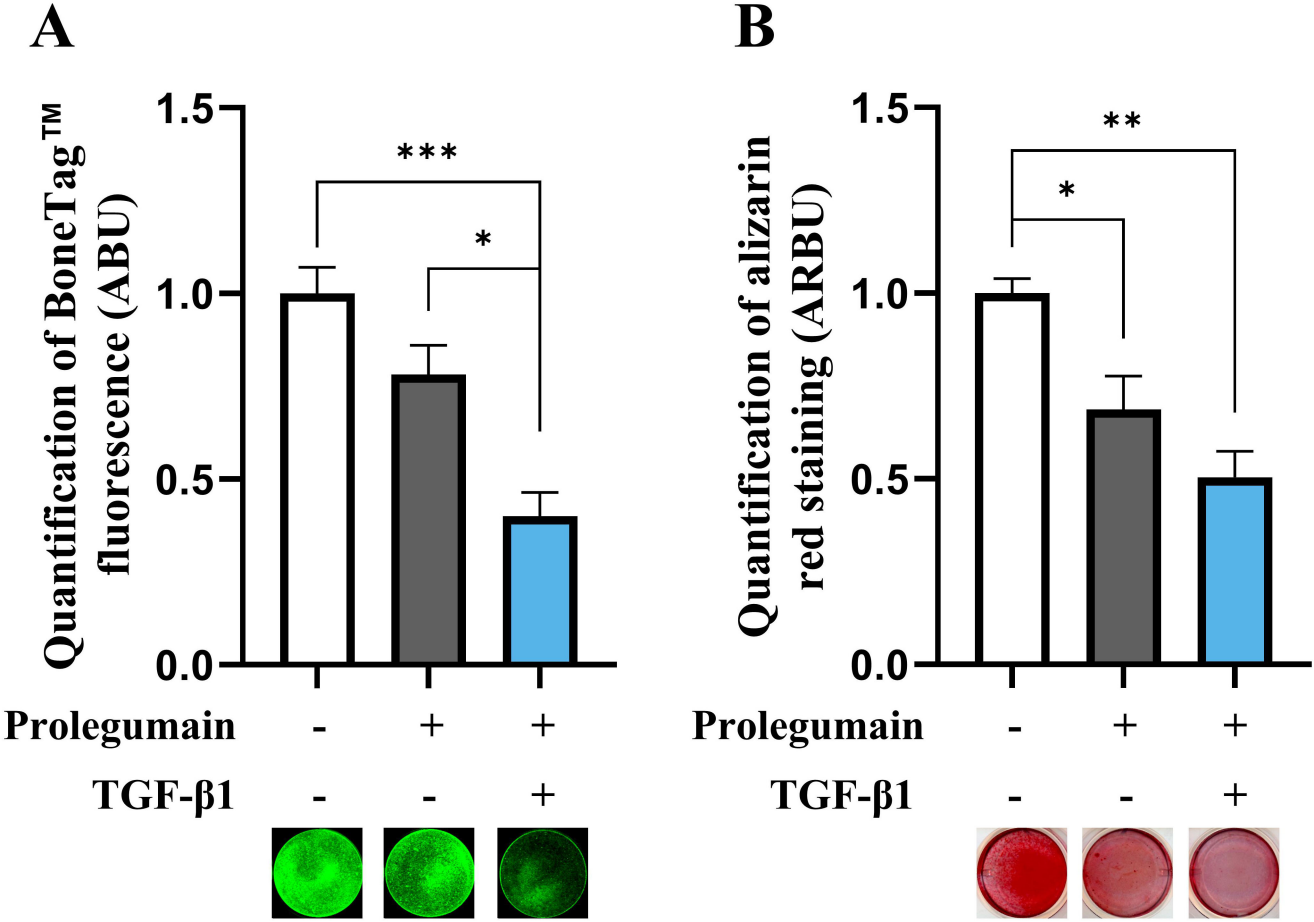
Inhibitory effect of pro-legumain on osteoblast maturation is exacerbated by TGF-β1. BMSC were cultured in osteoblast induction media for 14 days, either with 50% prolegumain-rich conditioned medium (165 ng/mL, control: 1.1 ng/mL) alone or in combination with TGF-β1 (25 ng/mL). Normalized matrix mineralization measured by BoneTag™ fluorescence [**(A)**, n=4] and alizarin red staining [**(B)**, n=3]. Data represent mean ± SEM. Two-way ANOVA (Šidák correction). *p<0.05, **p<0.01, ***p<0.001. Numbers (n) represent individual biological replicates.

After establishing that secreted prolegumain can be internalized and activated in osteogenic BMSC cultures, we examined if internalized prolegumain could act as a paracrine factor to inhibit matrix mineralization and if such inhibition was regulated by TGF-β1. BMSC were cultured for 14 days in osteogenic differentiation medium with or without legumain-rich condition media and with or without TGF-β1 (25 ng/mL). BoneTag™ fluorescence (**Figure 4A**) and alizarin red staining (**Figure 4B**) showed decreased matrix mineralization by treatment with prolegumain alone. In addition, concomitant treatment with prolegumain and TGF-β1 showed additive inhibition of mineralized matrix formation.

### 3.5 Pharmacological inhibition of legumain abolishes TGF-β1-induced inhibition of osteoblast maturation

**Figure 5:**
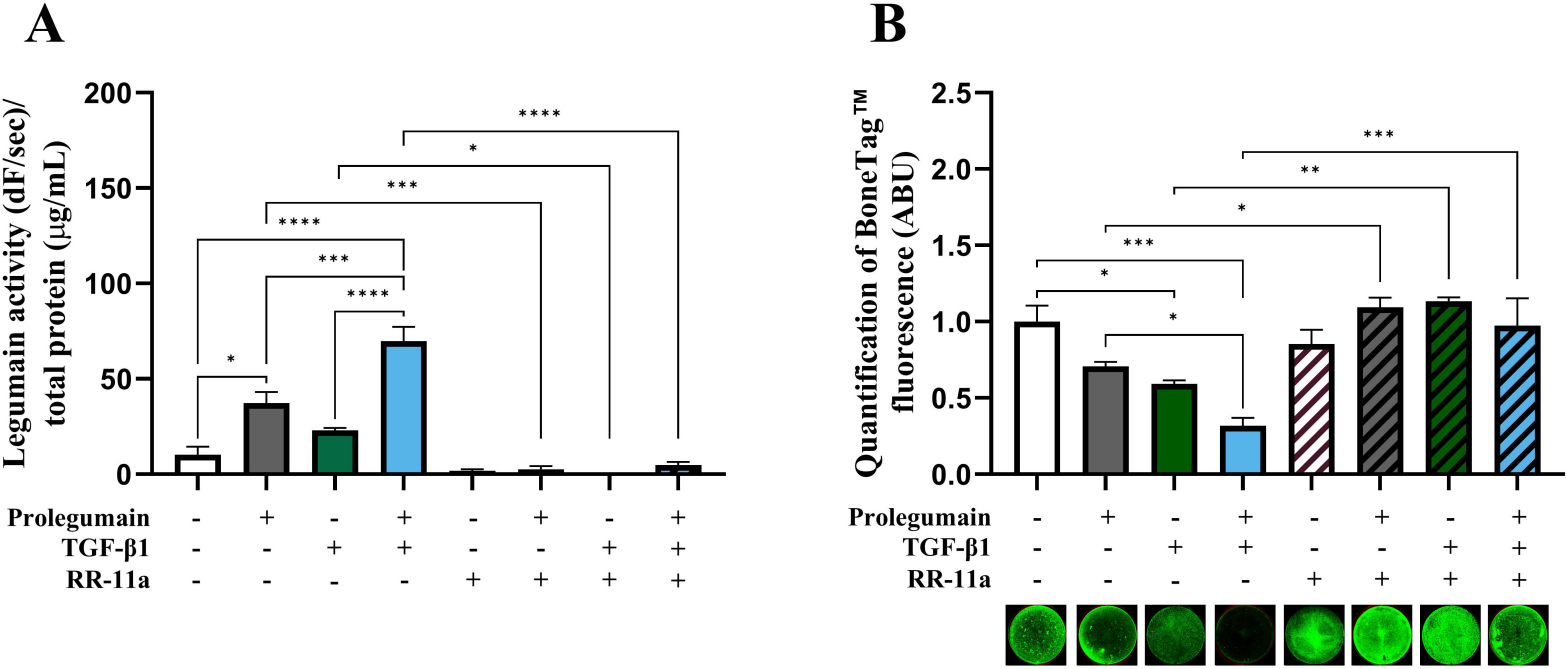
Pharmacological inhibition of legumain rescue matrix mineralization in osteogenic cells treated with TGF-β1 and prolegumain. BMSC were differentiated to osteogenic cells for 14 days with or without treatment with 50% prolegumain-rich conditioned medium (165 ng/mL, control: 1.1 ng/mL), TGF-β1 (25 ng/mL) and the legumain inhibitor RR-11a (50 μM). **(A)** Legumain activity (dF/sec) in cell lysates adjusted for total protein concentration (µg/mL) (n=3-4). **(B)** Normalized matrix mineralization measured by BoneTag™ fluorescence. Data represent mean ± SEM (n=3-4). Three-way ANOVA (Tukey’s *post-hoc*). *p<0.05, **p<0.01, ***p<0.001, ****p<0.0001.

Although presence of TGF-β1 in osteogenic BMSC cultures increased legumain levels associated with inhibited osteoblast maturation, this association could not prove that legumain mediated the inhibitory effect of TGF-β1 on osteoblast maturation. Therefore, we employed a selective, irreversible legumain inhibitor (RR-11a) and showed that presence of RR-11a (50 µM) in osteogenic BMSC cultures on day 14 significantly inhibited legumain activity, in the presence of legumain-rich media, TGF-β1 alone or concomitant treatment with both (**Figure 5A**). As expected, RR-11a also abolished the inhibitory effect of extracellular legumain on osteoblast maturation (**Figure 5B**). Interestingly, the TGF-β1-mediated inhibition of osteoblast maturation was completely abolished upon pharmacological inhibition of legumain by RR-11a. This data provides direct experimental evidence for the role of legumain in mediating the inhibitory effect of TGF-β1 on osteoblast maturation.

## 4 Discussion

Our study reveals a novel regulatory mechanism of osteogenesis, indicating that TGF-β1 modulates osteoblast maturation through the cysteine protease legumain. As a cytokine that is highly expressed in bone, TGF-β1 is postulated to be synthesized and deposited in the bone matrix in a latent form by osteoblasts during bone formation. It is then released and activated during bone resorption, playing a pivotal role in coupling bone resorption and formation. Extensive literature on the effects of TGF-β1 on skeletal homeostasis presents diverse and occasionally conflicting conclusions [reviewed in (24, 25)]. This variability in findings can partially be attributed to differences in experimental design and conditions across various research groups. Despite these differences, it is broadly accepted that TGF-β1 exerts a pleiotropic influence on osteogenic differentiation by promoting proliferation and early commitment to the osteoblastic lineage via non-canonical (Smad-independent) signaling pathways (25). On the other hand, it has been suggested that TGF-β1 inhibits osteoblast maturation through canonical (Smad-dependent) TGF-β signaling, as detailed by (25). While the mechanisms behind the promoting effect of TGF-β1 on early osteoblast differentiation are well-established, the mechanisms underlying its inhibitory effect on late-stage osteoblast differentiation (i.e. osteoblast maturation) remain poorly understood.

Our study demonstrates that TGF-β1 regulates legumain at different levels to modulate osteoblast maturation. We observed that TGF-β1 enhanced legumain activity and secretion in osteogenic cultures, despite a decrease in legumain mRNA expression. This suggests a possible post-transcriptional regulation of legumain by TGF-β1, potentially involving protein stabilization or increased processing of prolegumain to the mature form. Furthermore, we observed that osteogenic cells can internalize and activate extracellular prolegumain, leading to increased intracellular legumain activity. Interestingly, TGF-β1 treatment promoted the processing and activation of internalized prolegumain.

The concentration of TGF-β1 used in this study (25 ng/mL) was selected based on its clear inhibitory effect on osteoblast maturation, while maintaining (patho)physiological relevance, as the circulating levels of TGF-β1 in postmenopausal osteoporosis, a disease characterized by inhibited osteoblast differentiation and bone formation, has been shown to be 23.8 ng/mL, as compared to 15.6 ng/mL in age-matched control individuals (12). However, given the local release of TGF-β1 from the bone matrix during osteoclastic bone resorption, it is plausible that TGF-β1 concentration within the bone microenvironment is significantly higher. Further investigations are required to accurately quantify TGF-β1 levels within the bone microenvironment.

We have previously shown that legumain inhibits osteoblast maturation through an autocrine mechanism (16). The discovery that legumain is present in the secretome of various cell types and exists in extracellular compartments has spurred speculation about its potential paracrine regulatory roles (15). Here, we provide evidence indicating that extracellular prolegumain is internalized and activated in BMSC and during the early stages of osteogenic differentiation, similar to what we have previously described in kidney (HEK293) cells (18), although the legumain protein and activity levels returned to baseline in a time-dependent manner. Additional studies using e.g. labelled prolegumain could provide further evidence for internalization of the exogenous prolegumain in BMSC cultures and exclude the role of other possible events such as altered legumain mRNA stability or translation, or decreased degradation of endogenous legumain in the presence of prolegumain-rich media.

We observed that extracellular prolegumain inhibited osteoblast maturation, evidenced by decreased formation of mineralized matrix. Therefore, our findings provide novel experimental evidence that legumain acts as a paracrine regulator of osteoblast differentiation and function. However, additional studies are warranted to examine the *in vivo* relevance of our findings, although our studies are based on the well-established hBMSC-TERT cell line (26), which has previously been shown to be a suitable model for identifying molecular mechanisms regulating bone formation *in vivo* (16, 21).

Interestingly, we observed that extracellular prolegumain and TGF-β1 exert an additive effect on inhibition of osteoblast maturation. This observation was in line with our findings that TGF-β1 increases legumain secretion, internalization, and activation, but did not necessarily prove that legumain mediates the inhibitory effect of TGF-β1 on osteoblast maturation. Therefore, we used pharmacological inhibition of legumain in osteogenic cultures and observed that the inhibitory effect of extracellular prolegumain and TGF-β1 was completely abolished upon legumain inhibition by RR-11a. This interesting observation provides strong evidence for the inhibition of osteoblast maturation by TGF-β1 being mediated through the proteolytic activity of legumain. RR-11a is a selective, irreversible legumain inhibitor with no activity towards closely related proteases like caspases (27). In addition, currently there are not any reports indicating inhibition of other protases by RR-11a.

In the present study, we observed that TGF-β1 promoted activation of internalized prolegumain during osteoblast differentiation. Mature legumain will rapidly denature in non-lysosomal conditions (pH > 6) (28), indicating that prolegumain is processed and activated in the endolysosomal system after internalization. TGF-β1 alone did not have a direct effect on the expression of endogenous legumain. This indicates that the observed increase in mature legumain after combined treatment with TGF-β1 and prolegumain-rich medium was due to enhanced internalization and activation. However, it is possible that TGF-β1 alters the stability of endogenous legumain. Additional studies are warranted to investigate the mechanisms of TGF-β1 action on legumain internalization and activation. In most contexts, the process and significance of the internalization and subsequent activation of lysosomal proteases remain largely unexplored. Investigating the mechanism of legumain internalization was beyond the scope of the present work. However, we have previously shown that internalization of prolegumain is mediated by mechanisms independent of dynamin and plasma membrane cholesterol (29). TGF-β1 has been reported to promote autophagy in several cancer types (30, 31), presumably in a Smad and JNK-dependent manner (32). In addition, increased lysosomal activity has been demonstrated to have an important role in cellular remodeling during epithelial-to-mesenchymal transition (EMT) (33), an oncogenic process of which TGF-β1 is a major driver. Therefore, it is possible that the promoting effects of TGF-β1 on legumain secretion, internalization, and activity contribute to promotion of autophagy and increased lysosomal activity as observed in EMT.

Our study provides novel insight into the complex regulatory network governing osteoblast maturation unveiling a novel regulatory axis involving TGF-β1 and legumain. By demonstrating the role of legumain in TGF-β1-induced inhibition of osteoblast maturation, our study has important implications for bone disorders associated with aberrant TGF-β1 signaling and suggests legumain as a potential target for therapeutic interventions. Future studies are warranted to further explore the detailed mechanisms by which TGF-β1 regulates legumain secretion and function. Additionally, investigating the *in vivo* relevance of this pathway using animal models of bone diseases will be crucial for assessing its translational potential.

## 5 Conclusion

Legumain was shown as a paracrine regulator of osteoblast maturation. In addition, increased activation of endogenous and exogenous legumain by TGF-β1 was demonstrated. An additive inhibitory effect on osteoblast maturation was shown by concomitant treatment with TGF-β1 and prolegumain. Complete rescue of TGF-β1-induced inhibition of osteoblast maturation was demonstrated by pharmacological inhibition of legumain in cells concomitantly treated with prolegumain and TGF-β1, providing evidence that the inhibitory effect of TGF-β1 is mediated through legumain activation.

## Data Availability

The raw data supporting the conclusions of this article will be made available by the authors, without undue reservation.
